# Supplementary material for: Evolution of a Eukaryotic Transcription Factor’s co-TF Dependence Involves Multiple Intrinsically Disordered Regions Affecting Activation and Autoinhibition
Source: bioRxiv. 2024 Aug 29:2024.04.20.590343. Preprint. [Version 2] doi: 10.1101/2024.04.20.590343 (PMC11383300; doi:10.1101/2024.04.20.590343)
Supplement: Supplementary file 1 [file NIHPP2024.04.20.590343v2-supplement-1.pdf]

bioRxiv preprint doi: https://doi.org/10.1101/2024.04.20.590343; this version posted August 29, 2024. The copyright holder for this preprint (which was not certified by peer review) is the author/funder, who has granted bioRxiv a license to display the preprint in perpetuity. It is made available under aCC-BY 4.0 International license.

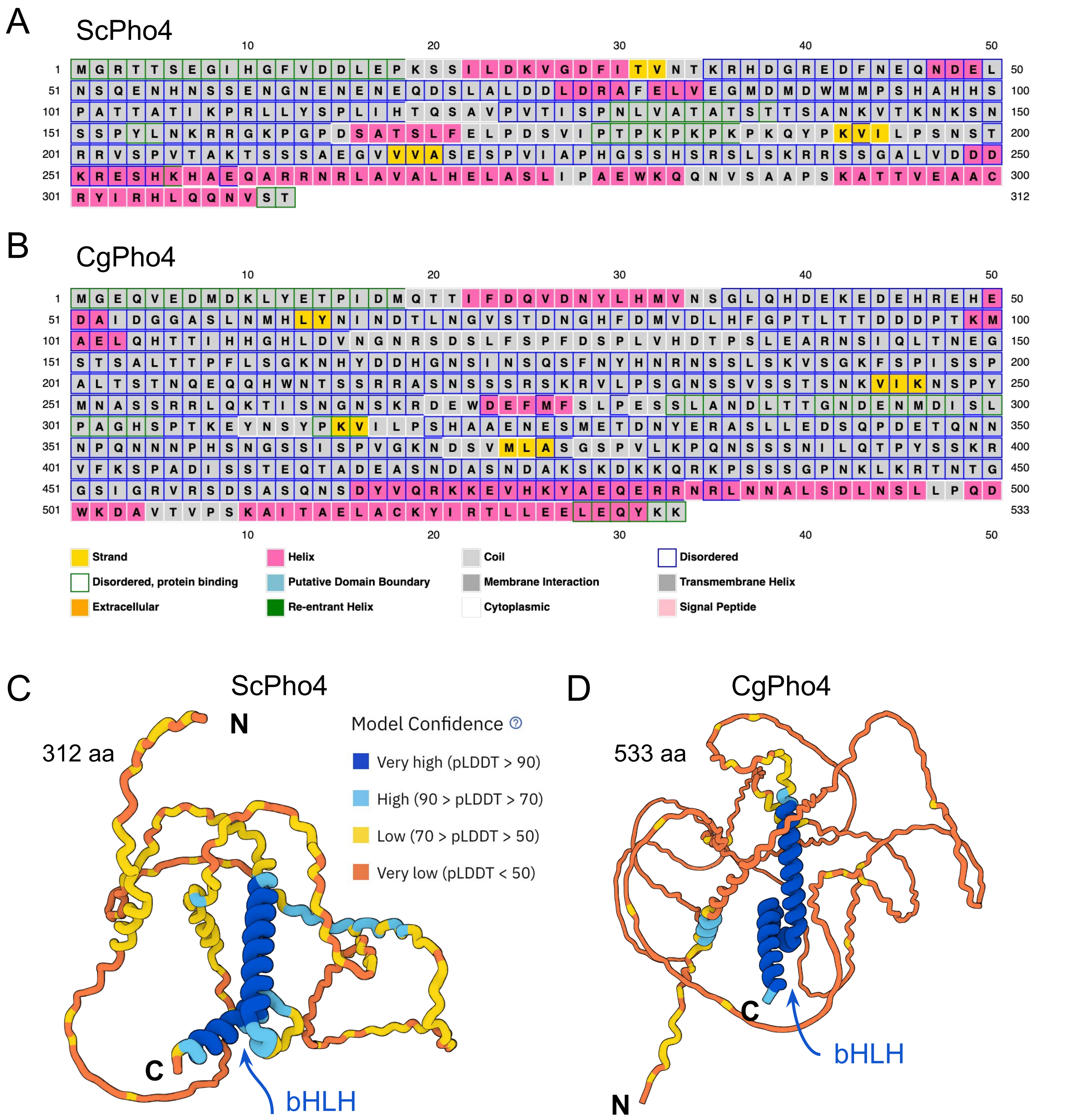

Fig. S1 ScPho4 and CgPho4 structural prediction shows both proteins are largely disordered outside the DBD. (A, B) Secondary structure predictions for ScPho4 and CgPho4 using the PSIPRED 4.0 server. (C, D) AlphaFold2 predicted monomer 3D structure for ScPho4 and CgPho4. Color represents model confidence. It has been shown that low and very low confidence regions overlap significantly and are predictive of protein disorder.

bioRxiv preprint doi: https://doi.org/10.1101/2024.04.20.590343; this version posted August 29, 2024. The copyright holder for this preprint (which was not certified by peer review) is the author/funder, who has granted bioRxiv a license to display the preprint in perpetuity. It is made available under aCC-BY 4.0 International license.

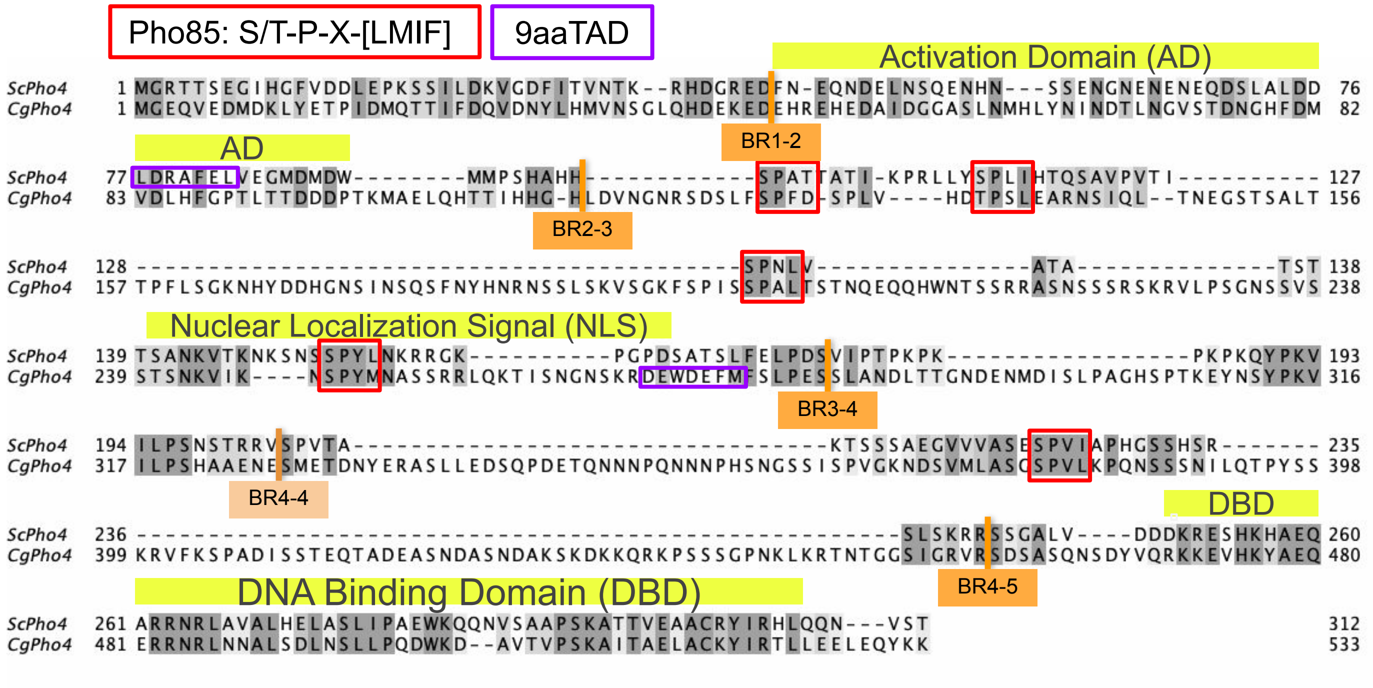

Fig. S2 ScPho4 and CgPho4 alignment and annotation. Protein sequence alignment was generated using ProbCons with 2 passes of consistency transformation and 100 iterative refinement, and was manually edited to align the known Pho85 recognition motifs (red boxes). The shading behind the amino acid symbols represent level of conservation based on BLOSUM62 scores. The functional annotation on the top were based on ScPho4 (Uniprot #P07270). The orange boxes and texts refer to the five breakpoints used for constructing chimeric Pho4 constructs (Fig. 5, 6).

bioRxiv preprint doi: https://doi.org/10.1101/2024.04.20.590343; this version posted August 29, 2024. The copyright holder for this preprint (which was not certified by peer review) is the author/funder, who has granted bioRxiv a license to display the preprint in perpetuity. It is made available under aCC-BY 4.0 International license.

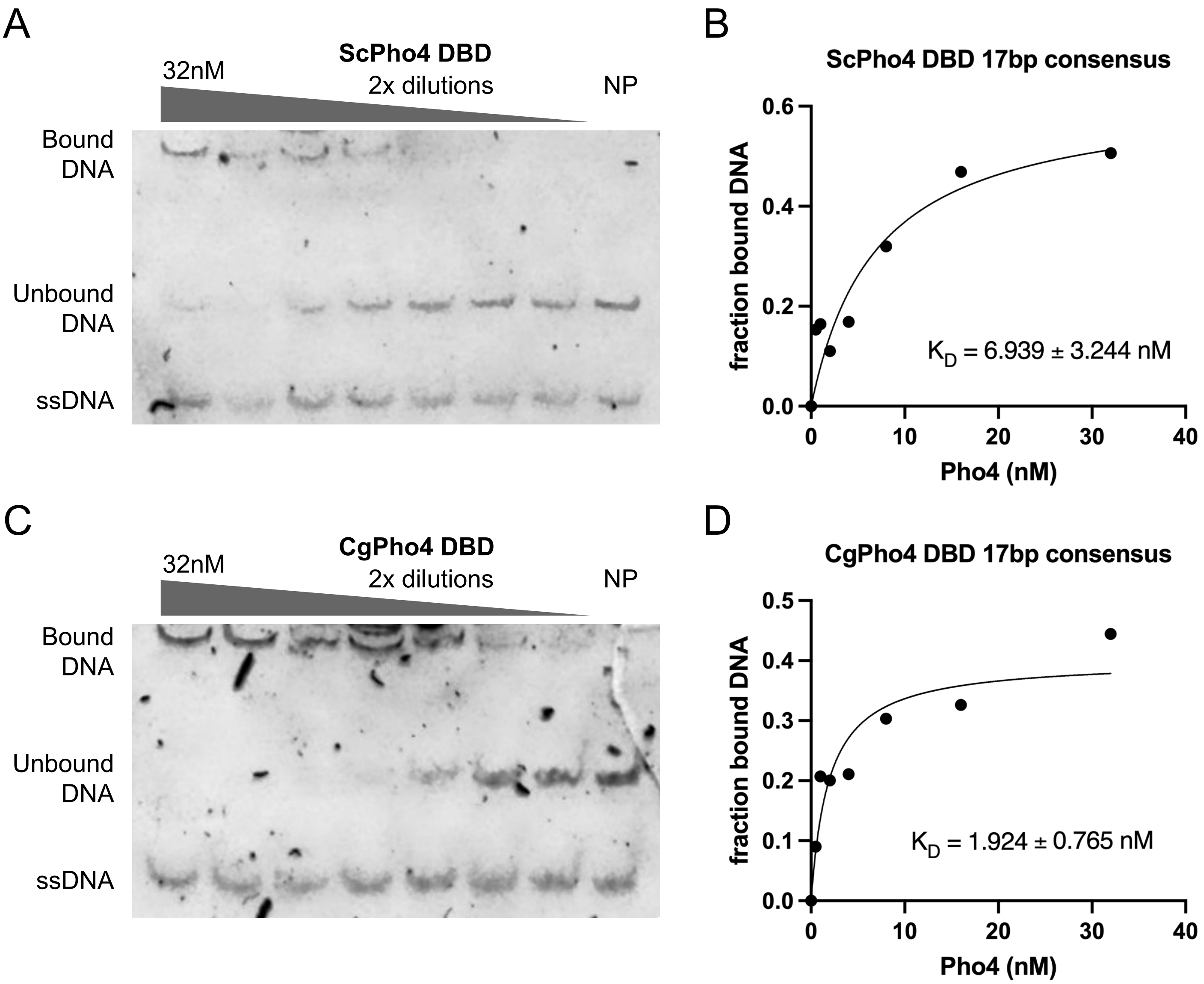

Fig. S3 Validation of difference in binding affinity to consensus motif. (A) EMSA with ScPho4 DBD and IR700 labeled 17bp consensus DNA. Lanes 1-7 contain 2x diluted protein; lane 8 is a no protein (NP) control. (B) Fraction of bound DNA was calculated from the unbound DNA band normalized to the NP lane. Fitting was done with a one site specific binding model in GraphPad Prism v10. C) EMSA with CgPho4 DBD and IR700 labeled 17bp consensus sequence. D) Same as (B) for CgPho4 DBD.

bioRxiv preprint doi: https://doi.org/10.1101/2024.04.20.590343; this version posted August 29, 2024. The copyright holder for this preprint (which was not certified by peer review) is the author/funder, who has granted bioRxiv a license to display the preprint in perpetuity. It is made available under aCC-BY 4.0 International license.

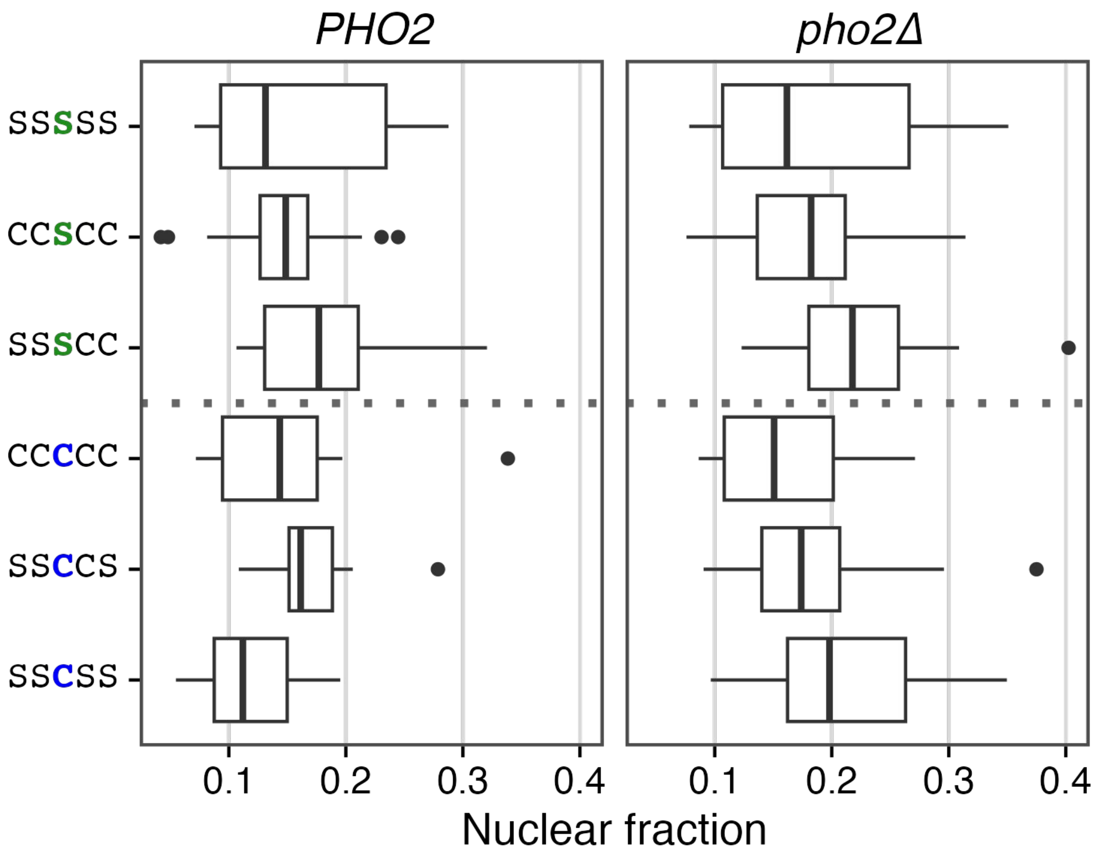

Fig. S4 NLS from ScPho4 and CgPho4 conferred similar levels of nuclear localization. Six Pho4 constructs with either NLS:Sc or NLS:Cg were quantified for their nuclear fraction in either *PHO2* or *pho2*Δ host background. Live fluorescence imaging was performed on mid-log phase cells. DAPI staining was used to identify the nucleus. Endogenous or chimeric Pho4 protein levels were quantified by the C-terminal mNeon tag. Integrated signal intensity inside the nucleus was divided by total integrated intensity across the entire cell for 35 cells in each construct. ANOVA with the identity of the NLS ("Cg" or "Sc") and Host (*PHO2* or *pho2*Δ) found the first factor to be not significant (F-test raw P = 0.13), i.e., no evidence for significant differences in nuclear fraction between constructs with NLS:Sc vs NLS:Cg. There was a significant difference for the second factor (F-test raw P = 0.0003), showing higher nuclear fraction in the *pho2*Δ background than in the *PHO2* background.
